# Incidence and predictors of virological failure among children receiving first-line anti-retroviral treatment in public comprehensive specialized hospitals found in Northeast Ethiopia: a retrospective follow-up study

**DOI:** 10.3389/fped.2024.1249957

**Published:** 2024-03-07

**Authors:** Estifanos Belay Abebe, Meseret Ekubay Gebregeorgis, Fuad Ahmed Seid, Alemu Birara Zemariam, Tadesse Mamo Dejene, Seteamlak Adane Masresha

**Affiliations:** ^1^ Department of Pediatrics Health, Woldia Comprehensive Specialized Hospital, Woldia, Ethiopia; ^2^ Department of Public Health, Debre Berhan University, Debre Berhan, Ethiopia; ^3^ Department of Nursing, Debre Berhan University, Debre Berhan, Ethiopia; ^4^ Department of Nursing, Woldia University, Woldiya, Ethiopia; ^5^ Department of Public Health, Woldia University, Woldiya, Ethiopia

**Keywords:** incidence, first-line, virological failure, children, Ethiopia

## Abstract

**Background:**

Despite anti-retroviral treatment coverage in resource-limited countries being highly appreciated, the occurrence of first-line virological failure remains a priority agenda. Therefore, this study serves as an input for evidence of virological failure among children.

**Objective:**

This study aimed to assess the incidence and predictors of virological failure among children receiving first-line anti-retroviral treatment in public comprehensive specialized hospitals found in Northeast Ethiopia through a retrospective follow-up study.

**Methods:**

A multicenter institution-based retrospective follow-up study was conducted on the medical records of 481 human immunodeficiency virus (HIV)-infected children who were on first-line anti-retroviral therapy from 1 January 2017 to 31 December 2021. Data were retrieved from 15 May to 15 June 2022 at three public comprehensive specialized hospitals. Study participants were recruited using a simple random sampling technique. STATA-14 was used to analyze the data, which was entered using EpiData version 4.6.2.0. The Kaplan–Meier estimator was used to estimate the survival. Both bivariable and multivariable Cox regression models were fitted to identify predictors. Finally, adjusted hazards ratios (AHRs) with 95% confidence intervals (CIs) were computed, and variables with a *P*-value of <0.05 were considered statistically significant predictors of virological failure.

**Result:**

A total of 481 children records were included in the final analysis, with an observed follow-up period of 16,379 person-months. Among these, 60 (12.47%) had developed virological failure, resulting in an overall incidence density rate of 3.67 (95% CI; 2.84, 4.73) per 1000 person-month observations. The hazards of virological failure (VF) among children were found to be increased by being in recent WHO stages III and IV (AHR = 3.688; 95% CI: 1.449–6.388), poor adherence to anti-retroviral treatment (ART) (AHR = 3.506; 95% CI: 1.711–7.234), and living in a rural environment (AHR = 5.013; 95% CI: 1.958–8.351). Conversely, the hazard of VF was reduced by 60% when the age of caregivers was less than 40 years (AHR = 0.405; 0.003–0.449).

**Conclusion and recommendations:**

The incidence rate of virological failure was relatively high. Living in a rural area, poor adherence to ART, being in a recent advanced WHO clinical stage, and having a caregiver of 40 years of age or older were all independent predictors of virological failure in children. Patients or parents (caregivers) need to be aware of the importance of strictly adhering to treatment regimens to prevent virological failure.

## Introduction

Human immunodeficiency virus (HIV) still remains a major public health concern around the world, causing acquired immunodeficiency syndrome (AIDS) and posing a significant threat to human survival (1). In 2021, 1.7 million children 0–14 years of age were living with HIV globally, with more than 90% residing in the sub-Saharan Africa region (2). In Ethiopia, approximately 42,000 children aged 0–14 years are living with HIV (3). Worldwide, 73% of all people living with HIV had access to anti-retroviral treatment (ART), as did 54% of children aged 0–14 years in 2020 (4). The Ethiopian Government launched fee-based ART in 2003, followed by free ART service in 2005 (5). Approximately 15,183 children under the age of 15 years were receiving ART in Ethiopia in 2021 (4).

Human immunodeficiency virus treatment failure is a suboptimal or non-sustained response to ART, which can be determined using clinical, immunological, or virological criteria, either alone or in combination (6). Virological failure (VF) is a plasma viral load (VL) of greater than 1,000 copies/mL based on two consecutive VL measurements after 3 months of adherence support and taking ART for at least 6 months. The hallmark of successful HIV treatment in children is virologic suppression (7). The World Health Organization (WHO) recommends VL monitoring to ensure VL suppression is achieved and maintained, but there are significant gaps in global access to VL monitoring, particularly in low- and middle-income countries, due to limited laboratory facilities and trained personnel (8). Maintaining long-term viral suppression among children on ART is very challenging, and their disease progression is also very rapid with poor outcomes (7, 9). Failure to detect VF early and continuation of the failing regimen may result in the gradual increment of resistant viruses, leading to clinical deterioration and death (10, 11).

Different studies reported the prevalence of VF, being 19.2% in South Africa (12), 25.5 in Cameroon (13), 64% in Senegal (14), 37% in Kenya (15), 23% in Asia (16), 29.2% in Iran (17), and 26% in the Netherlands (18). Cross-sectional studies conducted in resource-limited countries have reported a risk of VF ranging from 11% to 66% (19–21), and a 3-year probability of VF in Ghana was found to be 31% (22). A study conducted in Ethiopia showed that the incidence of VF in pediatrics was 18.3%, indicating a high burden of VF among the pediatric population (23).

Previously, different studies have identified various factors associated with VF, such as male gender (24), poor adherence (20), younger age (25), baseline CD4 count (26), clinical stage (21), and nevirapine-based regimen (17).

Despite efforts by the World Health Organization (WHO) and UNAIDS to increase the coverage of ART and achieve VL suppression, VF still remains a major public health concern among HIV-infected children (27), and their VL status is an under-recognized issue that receives poor attention in the field of pediatrics and within HIV/AIDS programs (28). The majority of studies conducted in resource-limited countries, including Ethiopia, are based on a combination of clinical, immunological, or virological criteria (29–35). Clinical and immunological criteria were the least sensitive (70%) and specific (46.7%) measures (36, 37). Since most of the available data in Ethiopia are from adult HIV-infected patients, extrapolating this to children is not possible as long as the drug has no similar therapeutic and side effects on the two populations (38). Nowadays, relying on clinical and immunological criteria to detect treatment failure is insufficient (39, 40). The World Health Organization (WHO) also recommends VL monitoring as the preferred tool for diagnosing and confirming failure (7, 41). Hence, this study intended to assess the incidence and predictors of VF in HIV-infected children receiving first-line ART in selected public comprehensive specialized hospitals in the Amhara region, Northeast Ethiopia.

## Materials and methods

### Study setting, design, and participants

The study was conducted in Comprehensive Specialized Hospitals (CSHs) located in the East Amhara region of Northeast Ethiopia, specifically in Debre Berhan, Dessie, and Woldia CSH. It was a multicenter retrospective follow-up study conducted among children who started ART from 1 January 2017 to 31 December 2021. Data were retrieved from 15 May to 15 June 2022. All HIV-infected children aged less than 15 years who had been on first-line ART for at least 6 months from 1 January 2017 to 31 December 2021 in selected CSH were included. Study participants with incomplete information like the date of ART enrollment and the date of VL measurement were excluded. The sample size was calculated using a single proportion formula with a proportion of VF of 5.1% (35), a 95% confidence level, and a 2% marginal error following a survival sample size calculation power approach tried with STATA 14.1 software. The final sample size of 512 HIV-infected children was determined, with an additional 10% adjustment for incomplete patient records. Initially, the medical record numbers of children under 15 years of age enrolled in ART from 1 January 2017 to 31 December 2021 were extracted from the electronic database of each hospital. The total number of children enrolled in ART at the three hospitals was 688. After that, the sample size was proportionally allocated to each hospital. Finally, using this database as a frame, a simple random sampling technique (computer-generated random number) was employed to recruit 512 sample records.

### Data collection

The data abstraction tool was adapted from the Ethiopian Federal Ministry of Health HIV/AIDS care and treatment follow-up forms. Charts were accessed based on their medical record numbers. Data were collected after having 1-day training for data collectors and supervisors. At each study location, a preliminary examination of 5% of the sample was conducted to determine whether the prepared checklists reflected the contents of the real study. The analysis did not incorporate data from the preliminary review. The outcome of this study was the incidence of virological failure, which was defined as a VL above 1,000 copies/mL based on two consecutive VL measurements taken after 6 months of ART initiation, with 3 months of enhanced adherence support following the first VL test (42). The first-line antiretroviral regimen in children of 3 or more years comprises two nucleoside analog reverse transcriptase inhibitors (NRTIs) as a backbone and one non-nucleoside reverse transcriptase inhibitor (NNRTI), while for children younger than 3 years comprises two NRTIs and a protease inhibitor (PI)-based regimen as the preferred choices (10). In this study, children were defined as individuals aged less than 15 years old (43). The survival time was the duration in months from the start of first-line ART to the development of VF. Children who were lost from HIV care and treatment, transferred out to other health facilities, died before developing VF during the follow-up time, or did not develop VF by the end of the study period were considered censored observations. Adherence to ART medications was classified as good, fair, and poor according to the percentage of drug dosage calculated from the total monthly dose of ART drugs as follows: Good (equal to or greater than 95% adherence or ≤3 doses missed per month), fair (85%–94% adherence or 4–9 dosesmissed per month), or poor (less than 85% adherence) (7, 42). The last date of follow-up was determined as the date when the child develops VF, if applicable, or the last date of VL test measurement conducted. Sociodemographic characteristics of the children (age, sex, residency and disclosure status, age of the caregiver, relationship with the caregiver, occupation, and marital status of the caregiver), clinical characteristics (baseline nutritional status, baseline and recent WHO clinical stage, baseline and recent CD4 count, and baseline and recent opportunistic infection), and antiretroviral medication-related factors [drug adherence, ART regimen type, ART regimen change, and prophylactic treatment with isoniazid (IPT) and cotrimoxazole (CPT)] were assessed.

### Data processing and analysis

The collected data were entered into EPI data version 4.6.2 statistical software before being exported to Stata version 14 for further analysis, and the data were checked for completeness and consistency, and then, the data were coded and cleaned. Multicollinearity between the candidate variables was checked by the variance inflation factor (mean VIF = 2.35). The Kaplan–Meier estimator was used to estimate the survival time and failure estimates. The fitness of the proportional hazards regression model was checked using the Schoenfeld residuals test (with global chi-square = 12.38, *P*-value = 0.8598) together with the graphical test. The assumptions were met for both tests. The Nelson–Aalen cumulative hazard rate relative to Cox–Snell residuals was used to validate the fitness model, and the Nelson–Aalen hazard function followed the 45-degree line very closely. *P*-values <0.25 in the bivariable analysis were entered into the multivariable analysis, and an adjusted hazards ratio (AHR) with a 95% confidence interval (CI) and a *P*-value <0. 05 was considered statistically significant.

## Results

### Sociodemographic characteristics of the child and child's caregiver information

In this retrospective follow-up study, a total of 512 medical records of children on ART were retrieved. Of these, 31 charts were excluded based on the exclusion criteria, and the remaining 481 charts of the children were included in the final analysis, yielding a card completion rate of 94%. Of the study participants, more than half (55.51%) of the children were boys; of these, 38 (14.2%) had VF. The median age of the children at the time of ART initiation was 8 years (IQR: 2.9, 13). The majority of research participants (60.9%) were from rural areas, and nearly one-fourth (23.1%) of the children were under the age of 5 (Table 1).

**Table 1:** Distribution of sociodemographic characteristics among HIV-infected children in public comprehensive specialized hospitals of the Amhara region, Northeast Ethiopia, 2022 (*n* = 481).

Variables	Category	Virological failure	
		Yes: *N* (%)	No: *N* (%)
Age	<5 years old	22 (19.8)	89 (80.2)
	5–10 years old	27 (10.1)	241 (89.9)
	>10 years old	11 (10.8)	91 (89.2)
Sex	Male	38 (14.2)	229 (85.8)
	Female	22 (10.3)	192 (89.7)
Residence	Urban	6 (3.2)	182 (96.8)
	Rural	54 (18.4)	239 (81.6)
Current status of parents	Both alive	29 (14.6)	175 (85.4)
	Either dead	22 (8.7)	230 (91.3)
	Both dead	8 (33.3)	16 (66.7)
Age of the caregiver	<40 years old	13 (4)	316 (96)
	≥40 years old	47 (30.9)	105 (69.1)
Educational status of the caregiver	No education	15 (27.3)	40 (72.7)
	Primary school	23 (18)	105 (82)
	Secondary school	19 (8.3)	210 (91.7)
	College/university	3 (4.3)	66 (95.7)
Marital status of the caregiver (*N* = 479)	Married	43 (12.1)	311 (87.9)
	Widowed	6 (7.8)	71 (92.2)
	Divorced	10 (20.8)	38 (79.2)
Occupational of the caregiver	Unemployed	39 (20.6)	150 (79.4)
	Private and non-government	12 (8.3)	133 (91.7)
	Governmental	9 (6.1)	138 (93.9)
Disclosure to children	Yes	5 (35.7)	9 (64.3)
	No	55 (11.8)	412 (88.2)
Relation of caregiver to the child	Parent	52 (11.4)	404 (88.6)
	Other	8 (28)	17 (68)
HIV status of the caregiver	Reactive	53 (11.5)	408 (88.5)
	Non-reactive	7 (35)	13 (65)

*N*, frequency; %, percentage.

### Baseline clinical and immunological characteristics

Nearly 29% of the study participants were in advanced WHO clinical stages (III and IV) at ART initiation; of these, 27 (19.6%) experienced VF. During ART follow-up, the majority of children (94.8%) experienced opportunistic infections, and 56 (12.3%) of them developed VF (Table 2).

**Table 2:** Clinical and immunological characteristics of HIV/AIDS infected children in public comprehensive specialized hospitals of the Amhara region, Northeast Ethiopia, 2022.

Variables	Category	Virological failure	
		Yes: *N* (%)	No: *N* (%)
WHO stage at baseline	Early stage (I and II)	33 (9.6)	310 (90.4)
	Advanced stage (III and IV)	27 (19.6)	111 (80.4)
Baseline nutritional status (*N* = 194)	Normal	11 (8.8)	114 (91.2)
	Underweight	26 (50)	26 (50)
	Overweight	1 (5.9)	16 (94.1)
Baseline functional status for age >5 years (*N* = 369)	Working	1 (5.6)	17 (94.4)
	Ambulatory	39 (11.1)	312 (88.9)
Baseline opportunistic infections	Yes	56 (12.3)	400 (87.7)
	No	4 (16)	21 (84)

### Patient follow-up and ART-related characteristics

The majority and of children received CPT (67.6%) and IPT(64.7%); of these, 43 (13.3%) and 37 (11.9%) developed VF, respectively. Nearly one-third (35.6%) of children were in advanced WHO T-stages (III and IV); of these, 54 (31.6%) had VF. Nearly one-fourth (22.9%) had poor ART adherence; of these, 53 (46.5%) developed VF (Table 3).

**Table 3:** Patient follow-up ART-related characteristics of HIV/AIDS-infected children in public comprehensive specialized hospitals of the Amhara region, Northeast Ethiopia, 2022 (*n* = 481).

Variables	Category	Virological failure	
		Yes: *N* (%)	No: *N* (%)
History of regimen change	Yes	12 (48)	13 (52)
	No	48 (10.5)	408 (89.5)
ARV prophylaxis for PMTCT	Yes	2 (50)	2 (50)
	No	58 (12.2)	419 (87.8)
CPT prophylaxis	Yes	43 (13.3)	282 (86.7)
	No	17 (10.8)	140 (89.2)
INH prophylaxis	Yes	37 (11.9)	274 (88.1)
	No	23 (13.5)	147 (86.5)
Adherence to ART	Good	5 (1.6)	308 (98.4)
	Fair	2 (3.7)	52 (96.3)
	Poor	53 (46.5)	61 (53.5)
Recent WHO T-stage	Early stage (I and II)	6 (1.9)	304 (98.1)
	Advanced stage (III and IV)	54 (31.6)	117 (68.4)
Virological failure		60 (12.5)	421 (87.5)

### Incidence of VF among HIV-infected children on ART

Four hundred eighty-one HIV-infected children were followed for a maximum of 60 months, with a median follow-up of 41.8 months, and the total person-time observation was 16,379 person-months. The proportion of VF among HIV-infected children on first-line ART was 12.47% (95% CI: 4.80, 15.75), and the remaining 87.53% were censored observations (Table 3). The incidence density rate (IDR) of VF was 3.67(95% CI; 2.84, 4.73) per 1,000 person-months of observation. The estimated mean survival time using the restricted mean of the entire follow-up was 52.83 months (95% CI: 51.39, 54.28 months).

### Kaplan–Meier survival virological failure estimates of children among HIV-infected children on ART

**Figure 1:**
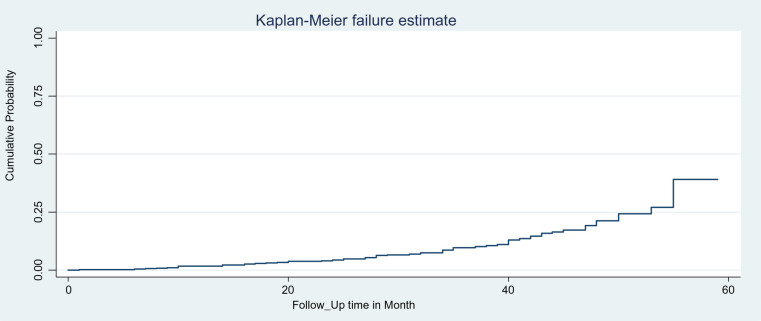
Overall Kaplan–Meier failure estimate of VF among HIV-infected children on first-line ART in the east amhara region, Northeast Ethiopia, 1 January 2017, to 31 December 2021.

The overall Kaplan–Meier estimates revealed that the probability of developing VF among children on first-line ART was low in the first months of starting the treatment, which relatively increased as the follow-up duration increased (Figure 1). The median age of children at ART initiation was 101 months (IQR: 60–109). During the first months and by the end of the follow-up period, the survival probability was 99.79% (95% CI: 98.49, 99.97) and 60.84% (95% CI: 51.87, 76.29), respectively.

### Predictors of virological failure among HIV-infected children on ART

In the bivariable Cox proportional hazard model, residence, the current status of the caregiver, age of the caregiver, caregiver occupation, HIV status of the caregiver, marital status of the caregiver, disclosure to children, baseline WHO stage, CPT, IPT, recent WHO T-stage, and adherence were significantly associated with the incidence of VF. However, in the multivariable Cox proportional hazard model, residence, age of the caregiver, ART adherence, and recent WHO T-stages (III and IV) remained significantly associated with VF.

Keeping other variables constant, the hazard of developing VF among children living in rural areas was five times (AHR = 5.013; 1.958–8.351) higher than those living in urban areas. The hazard of developing VF among children with poor adherence was 3.5 times (AHR = 3.506; 95% CI: 1.711–7.234) more likely than among children with good adherence.

Regarding the WHO clinical stage, the hazard of developing VF in a patient with advanced recent WHO T-stages III and IV was 3.7 times (AHR = 3.688; 95% CI: 1.449–6.388) higher than those who were in recent WHO T-stages I and II. In addition to this, the hazards of VF were lowered by 59.5% (AHR = 0.405; 95% CI: 0.003–0.449) when the age of the caregiver was less than 40 years (Table 4).

**Table 4:** Bivariable and multivariable Cox proportional hazard analysis result among HIV/AIDS infected children in public comprehensive specialized hospitals of Amhara Region, Northeast Ethiopia, 2022 (*n* = 481).

Variables	Variable category	CHR (95% CI)	AHR (95% CI)
Residence	Urban	1	1
	Rural	6.245 (0.587–3.196)	**5.013** **(1.958**–8.351)^*^
Current status of parents	Both alive	1	1
	Either dead	0.524 (0.301–0.910)	0.477 (0.197–1.157)
	Both dead	2.947 (1.347–6.444)	3.248 (0.178–59.222)
Age of the caregiver	<40 years	1.141 (0.375–3.466)	**0.405** (**0.003**–0.449)^*^
	>40 years	1	1
Marital status of the caregiver	Married	1	1
	Widowed	0.781 (0.331–1.841)	0.826 (0.238–2.869)
	Divorced	1.785 (0.896–3.557)	0.853 (0.318–2.284)
Occupation of the caregiver	Unemployed	3.708 (1.796–7.657)	2.370 (0.616–9.124)
	Private	1.345 (0.567–3.193)	2.752 (0.467–30.554)
	Governmental	1	1
HIV status of the caregiver	Reactive	1	1
	Non-reactive	3.625 (1.646–7.988)	2.87 (0.665–4.332)
Disclosure to children	Yes	1	1
	No	0.257 (0.102–0.643)	1.461 (0.394–5.416)
WHO stage at baseline	Early stage (I and II)	1	1
	Advanced stage	1.663 (0.996–2.778)	0.533 (0.241–1.176)
CPT use	Yes	1	1
	No	0.459 (0.254–0.828)	0.411 (0.119–1.416)
CPT use	Yes	1	1
	No	0.707 (0.411–1.216)	0.323 (0.112–1.940)
Adherence to ART	Good	1	1
	Fair	1.323 (0.693–2.527)	1.613 (0.762–3.412)
	Poor	8.972 (1.001–9.015)	**3.506** (**1.711**–7.234)^*^
Recent WHO stage	Early stage (I and II)	1	1
	Advanced WHO stage	7.220 (2.011–11.044)	**3.688** (**1.449**–6.388)^*^

Bold indicates statistically significant determinants.

^*^ *P* < 0.05.

## Discussion

This retrospective follow-up study aimed to assess the incidence and predictors of VF among HIV-infected children on ART in comprehensive specialized hospitals in the Amhara region of Northeast Ethiopia. The overall incidence density rate of VF was found to be 3.67 (95% CI: 2.84, 6.73) per 1,000 person-month observations, which was predicted by recent WHO T-staging, residence, ART adherence, and age of the caregiver. This study also revealed that the proportion of VF among HIV-infected children on ART was found to be 12.47% (95% CI: 4.80, 15.75).

The overall incidence obtained from this study was in line with studies conducted in southern Ethiopia (4.97) (26), Tigray region (5.1) (35), and Bahir Dar (14.8) (44). This similarity could be due to the use of a similar study design and cutoff point for defining VF. In addition, the HIV/AIDS prevention and control program of Ethiopia has recently given special attention to people living with HIV/AIDS in all areas to meet the national 95% targets, which may affect VF (21). Conversely, our finding was lower than studies conducted in Kenya (28%) (28), Cameroon (53%) (24), Uganda (38%) (45), and Bahir Dar (34%) (30). This disparity could be attributed to differences in the cutoff point used to define VF, inclusion criteria, and study year. In investigations conducted in Kenya and Cameroon, the presence of detectable virus in plasma at concentrations larger than 500and 200 copies/mL, respectively, was considered VF, which might increase the number of VFs in those studies (24, 28). In contrast, studies conducted in Bahir Dar and Uganda defined VF as a single increase in VL measurement above 1,000 copies/ml following treatment; this could increase the number of VFs (30, 45). Furthermore, studies conducted in Bahar Dar included children whose VL was requested into the laboratory during the study period. Furthermore, in a study conducted in Uganda, death after at least 6 months of treatment was considered as VF, which may potentially overestimate VF. Another explanation for this discrepancy could be the study period; for example, the current study was conducted 9 years after the Cameroon and Uganda studies, and since then, there have been numerous advancements in ART services, such as the availability of VL monitoring, which ultimately lowers the incidence of VF (21). This may be explained by the recent increase in the use of VL tests to track the efficacy of ART.

In terms of adherence, the current study found that children who had poor drug adherence to first-line ART regimens were 3.5 times more likely to experience VF than those who had good adherence. This finding is comparable with the studies conducted in Uganda and Malawi (20, 45), Kenya (15), southern Ethiopia (26), central Oromia (46), Cameron (24), and Tanzania (47). This is because optimal adherence is required to suppress VL and improve clinical and immunological outcomes (35). For HIV/AIDS-infected patients, ART is recommended to suppress VL, maintain high immunity, and prolong survival. However, the success of ART depends on drug adherence (42). Poor adherence reduces drug effectiveness, which reduces immunity, increasing the risk of opportunistic infection and drug resistance (44). This is because a high level of sustained adherence is necessary to suppress viral replication and improve immunological and clinical outcomes, which in turn decrease the risk of developing ART drug resistance and reduce the risk of VF. On the contrary, poor adherence to ART drugs is commonly encountered in the treatment of HIV-infected children due to a variety of factors. First, pediatric regimens sometimes call for the regular dosage of many medications, each of which has the potential to cause side effects and drug interactions. Second, characteristics specific to the liquid/capsule formulation and regimen, such as poor palatability, a high pill burden, short dosing intervals, and problems with medication storage, have a substantial impact on low ART adherence (48). This leads to increased rates of CD4 destruction, viral replication, buildup of drug-resistant viruses, and rapid disease progression, all of which are contributing factors to VF.

Following the initiation of therapy, children in WHO T-stages III and IV had 3.7 times higher risk of VF than those in WHO stages I and II, which agrees with findings from research conducted in Mozambique (20) and Uganda (9). This may be the result of severely compromised immune systems in children in advanced WHO clinical stages, rendering them unable to fight infections and increasing the likelihood of comorbidities and VL, which in turn increase the risk of VF.

Children in rural areas had 5.8 times higher risk of VF than children in urban areas in this study. This outcome is in line with research from Southern Ethiopia and South Africa (49, 50). This can be explained by the challenges associated with assessing healthcare facilities in rural areas, including long transportation times, lack of transportation, poor road conditions, and transport costs imposed on patients when visiting a health facility that is far from homesteads (9). This may result in poor medication adherence, which increases susceptibility to VF (49). Furthermore, those living in urban may have their follow-up closely monitored and may have more information about HIV/AIDS.

Regarding the age of caregivers, caregivers aged <40 years lowered the risk of VF by 59.5%. This result is supported by studies conducted in Ethiopia (51), Bahir Dar (44), and the Tigray region (35). This might be due to the fact that educational programs on HIV/AIDS have largely been targeted at young adults (52), which may help young caregivers to disclose their children's HIV status and may improve adherence and retention in care, as well as being protective against VF. The study revealed that older adults need additional education regarding HIV/AIDS (53). Older age may reduce cooperativeness to HIV care and treatment. This study highlighted that viral suppression may have been mediated by caregivers’ increased capacity to support children's treatment adherence.

This study indicates a delay in children's ART initiation age. High rates of late ART initiation in pediatric (53.2%) (54) and adult (39%) (55) populations nationwide provided proof for this claim. The study's greater proportion of rural inhabitants, the absence of HIV-positive family members (54, 55), the low financial status of families or caregivers, and the lack of awareness about the advantages of initiating HIV treatment early (56) could be the causes.

## Limitations and strengths of the study

This study has some important limitations that should be considered when interpreting the results. Because of the retrospective nature and incomplete medical records, some important predictors, such as baseline VL, socioeconomic status, and laboratory investigations, were excluded. The study was conducted at a multicenter healthcare facility and comprised 5 years of follow-up with a proportional distribution of the sample. Data were collected by experienced health professionals working at the ART care and support center, which significantly improved the quality of collected data.

## Conclusion and recommendations

The findings of this study indicate that there was a relatively high rate of VF among children. Residence, age of the caregiver, poor drug adherence, and advanced recent WHO clinical stage were found to be significant predictors of VF among HIV-infected children on first-line ART in comprehensive specialized hospitals of the Amhara region. Patients or parents (caregivers) need to be aware of the importance of strictly adhering to treatment regimens to prevent VF. Service providers should better consider addressing important variables for the occurrence of VF, such as being in a rural area, poor drug adherence, and advanced WHO clinical stage after ART initiation.

## Data Availability

The raw data supporting the conclusions of this article will be made available by the authors without undue reservation.
